# Cutting-edge insights into liver fibrosis: advanced therapeutic strategies and future perspectives using engineered mesenchymal stem cell-derived exosomes

**DOI:** 10.1007/s13346-024-01784-7

**Published:** 2025-01-24

**Authors:** Manar A. Didamoony, Ayman A. Soubh, Lamiaa A. Ahmed

**Affiliations:** 1 Pharmacology and Toxicology Department, Faculty of Pharmacy, Egyptian Russian University, Cairo, 11829 Egypt; 2 Pharmacology and Toxicology Department, Faculty of Pharmacy, Ahram Canadian University, 6th of October City, Giza, 12451 Egypt; 3 Pharmacology and Toxicology Department, Faculty of Pharmacy, Cairo University, Cairo, 11562 Egypt

**Keywords:** Exosomes, Exosome engineering, Mesenchymal stem cells, Liver fibrosis, Pathogenesis of liver fibrosis

## Abstract

Liver fibrosis is still a serious health concern worldwide, and there is increasing interest in mesenchymal stem cells (MSCs) with tremendous potential for treating this disease because of their regenerative and paracrine effects. Recently, many researches have focused on using the released exosomes (EXOs) from stem cells to treat liver fibrosis rather than using parent stem cells themselves. MSC-derived EXOs (MSC-EXOs) have demonstrated favourable outcomes similar to cell treatment in terms of regenerative, immunomodulatory, anti-apoptotic, anti-oxidant, anti-necroptotic, anti-inflammatory and anti-fibrotic actions in several models of liver fibrosis. EXOs are superior to their parent cells in several terms, including lower immunogenicity and risk of tumour formation. However, maintaining the stability and efficacy of EXOs after in vivo transplantation remains a major challenge in their clinical applicability. Therefore, several strategies have been applied in EXOs engineering, such as parental cell modification or modifying EXOs directly to achieve optimum performance of EXOs in treating liver fibrosis. Herein, we discuss the underlying mechanisms of liver fibrosis with an overview of the available therapies, among them EXOs. We also summarise the recent developments in improving the effectiveness of EXOs with the advantages and limitations of these approaches in terms of the upcoming clinical applications.

**Graphical Abstract:**

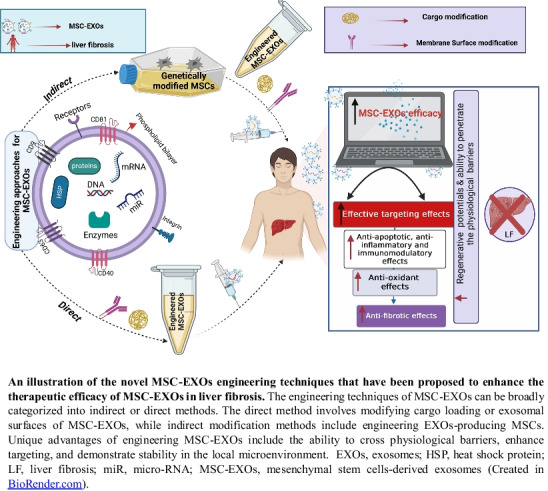

## Introduction

Hepatic fibrosis is a pathological process involving abnormal tissue proliferation in the liver resulting from various pathogenic factors like virus infections, poor nutrition, alcohol abuse, and hepatotoxins, and it may further develop into cirrhosis or even hepatocellular carcinoma in case of inappropriate intervention within a suitable time [1]. Worldwide, liver fibrosis is correlated with elevated mortality and morbidity rates, where more than one million people die every year from this disease, with an estimated 1.5 billion cases globally [2].

The core pathophysiological conflux point of multiple trajectories that eventually lead to liver fibrosis is the energizing of hepatic stellate cells (HSCs) [3]. HSCs proliferate and acquire morphological changes to become myofibroblast-like cells, which elaborate an excessive amount of extracellular matrix (ECM) such as collagen that accumulates in the liver, disrupting hepatic architecture and ultimately leading to liver failure [4]. Notably, increasing evidence shows that liver injury is associated with oxidative stress and inflammation as essential partners that present simultaneously and interact with each other, creating a vicious cycle leading to exaggerated hepatic fibrosis [3]. During this cycle, the necroptotic signalling is aberrantly triggered with the release of inflammatory cytokines such as high mobility group box 1 (HMGB1) protein, which stimulates the quiescent HSCs after inducing transforming growth factor-beta 1 (TGF-β1) release [5]. Over and above that, activated HSCs produce hedgehog (Hh) ligands that eventually activate the Hh pathway, which provokes hypoxia. In turn, hypoxia initiates an intracellular axis that links to hypoxia-responsive elements found in the promoter of several target genes, including vascular endothelial growth factor (VEGF), and increases its overexpression. Besides, VEGF directly stimulates liver sinusoidal endothelial cells (LSECs) and provokes pathological angiogenesis together with HSCs activation [6].

In the past two decades, stem cell therapy offered a glimmer of hope for treating liver fibrosis. Mesenchymal stem cells (MSCs) have considerable potential in regenerative medicine applications owing to their robust immunomodulatory and tissue repair capacities through homing, cell-cell interaction, and paracrine mechanisms [7]. In addition, multiple researches reported data estimating the dependence of MSCs’ biological effects on cytokines, chemokines, growth factors, and extracellular vesicles (EVs) released by these MSCs [8].

Exosomes (EXOs), microvesicles, and apoptotic bodies are the primary components of EVs. There is supported evidence that EXOs play significant roles in major biological processes, including disease and regeneration. In diseased conditions associated with fibrosis, EXOs act as essential mediators for intercellular communication, transporting cytokines, regulating immune responses, and affecting the trajectories associated with inflammation and fibrosis [9]. On the other hand, MSC-derived EXOs (MSC-EXOs) have the biological properties of MSCs and aid in tissue regeneration by transporting and encapsulating a wide variety of cytokines, growth hormones, signalling lipids, messenger RNAs (mRNAs), and microRNAs (miRs) [10]. In several experimental models of hepatic fibrosis, MSC-EXOs showed regenerative, immunomodulatory, anti-oxidant, anti-inflammatory, anti-apoptotic, anti-necroptotic, and anti-fibrotic properties [5, 11].

Accumulating evidence indicates that MSC-EXOs are as functional as, if not more so than, their parent cells [10, 12]. MSC-EXOs retain all the desirable traits passed down from their parents in addition to reduced immunogenicity, improved safety profile, increased drug delivery efficiency, improved intercellular communication range, and improved biocompatibility. Furthermore, there is no evidence that MSC-EXOs are carcinogenic or cause undesirable immune system reactions. The inability of EXOs to sustain their effectiveness and stability after in vivo transplantation is one of the main obstacles in their clinical use despite their encouraging potential. So, we need to look into novel methods, such as engineering strategies, to make EXOs more effective and stable upon their use [13, 14].

In this review, we first provided an overview of liver fibrosis and its recent underlying pathological mechanisms. We also spotlighted the novel potential therapies for liver fibrosis, which are among them EXOs. Besides, we highlighted the recent developments to improve the effectiveness of EXOs using engineering strategies for modifying their parent cells or directly the derived EXOs. Finally, we discussed the limitations and future perspectives of these approaches in the upcoming clinical applications.

## Pathogenesis of liver fibrosis

**Fig. 1:**
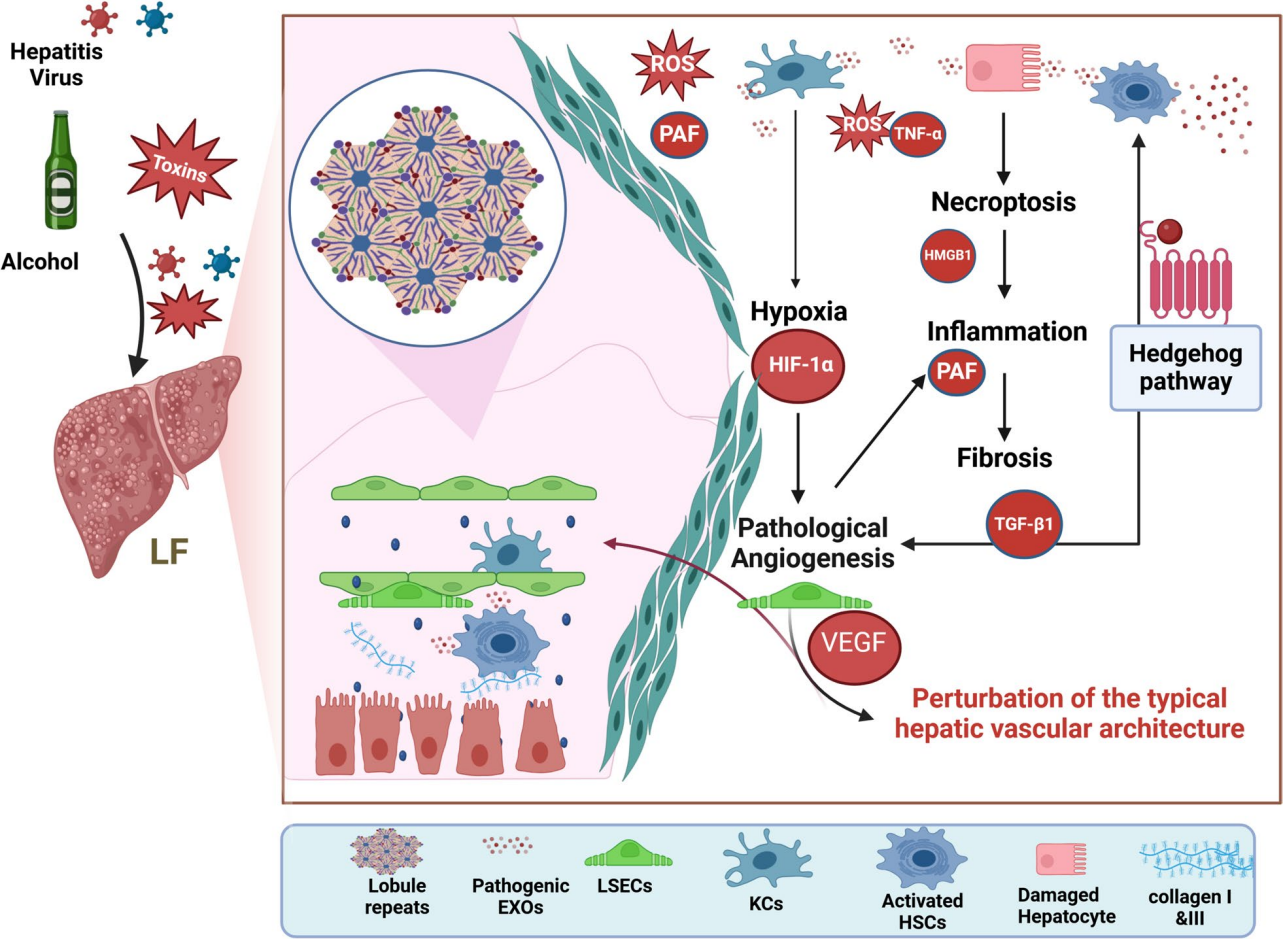
Pathogenic mechanisms of liver fibrosis, including interactions between hepatic parenchymal and non-parenchymal cells and a multitude of signalling pathways. Hepatocytes and parenchymal epithelial cells release inflammatory mediators to support the activation of HSCs. As a result, HSCs multiply and undergo morphological modifications to resemble myofibroblast-like cells. These cells then produce an excessive amount of ECM, including collagens I and III, which builds up in the liver and eventually causes hepatic architecture to be disrupted and liver failure to result. Specifically, damaged cells provoke oxidative stress and inflammatory responses as necessary partners that manifest concurrently and interact with one another, generating a vicious cycle that exacerbates hepatic fibrosis. Oxidative stress and inflammatory events during this cycle cause the release of inflammatory cytokines, such as HMGB1 protein, which stimulates quiescent HSCs after inducing TGF-β1 release, thereby initiating necroptotic signalling aberrantly and promoting LF. In addition, activated HSCs generate hedgehog ligands, which attach to their patched receptor and ultimately activate the hedgehog pathway, increasing HSCs activity and inducing hypoxia. Hypoxia, in turn, sets off an intracellular axis involving the stimulation of transcription factor HIF-1α, which is linked to regions in the promoter of multiple target genes, including VEGF, that are responsive to hypoxia and enhance its overexpression. Additionally, VEGF directly increases HSCs and LSECs activation, resulting in pathological angiogenesis. Furthermore, pathogenic EXOs play a crucial role in the pathogenesis of LF by controlling HSCs activation, and immune cell interaction as well as affecting the signalling pathways associated with inflammation and fibrosis. ECM, extracellular matrix HIF-1α, hypoxia-inducible factor-1alpha; HMGB1, high mobility group box-1; KCs, kupffer cells; LF, liver fibrosis; LSECs, liver sinusoidal endothelial cells; PAF, platelet-activating factor; ROS, reactive oxygen species; TGF-β1, transforming growth factor-beta 1; TNF-α, tumour necrosis factor-alpha; VEGF, vascular endothelial growth factor (Created in *BioRender.com*)

Liver fibrosis is a quintessential case of the liver’s universal response to acute or chronic liver injury. Crosstalk between parenchymal and non-parenchymal cells, as illustrated in Fig. (1), occurs during the progression of chronic liver injury toward fibrosis, resulting in the stimulation of diverse immune cells and signalling cascades. Apoptosis is induced in the injured hepatocytes, and LSECs endure sinusoid capillarization, which is the loss of fenestrae. Kupffer cells (KCs) are activated, resulting in the production of a diverse array of cytokines and chemokines [15]. Finally, quiescent HSCs are activated and express novel receptors and proteins, including the platelet-derived growth factor receptor, TGF-β1 receptor, and alpha-smooth muscle actin (α-SMA). The proliferation and production of ECM proteins by activated HSCs result in the formation of a fibrous scar [16]. Besides, most of the fibrotic tissue is found near the portal tracts, increasing the resistance to blood flow and hepatocellular dysfunction due to excess collagen accumulation [17].

### The central signalling cascades during liver fibrosis

HSCs activation is a critical pathogenic event in hepatic fibrosis. Following HSCs activation, a complex series of events occurs through the induction of signalling cascades that essentially regulate the fibrotic response of the HSCs during hepatic fibrosis [18]. Below is a description of several signalling cascades that have been investigated in the pathogenesis of hepatic fibrosis.

### Oxidative stress in liver fibrosis

One of the vital pathophysiological components of hepatic fibrosis is oxidative stress [19]. Long-term chronic exposure to harmful agents such as hepatitis viruses and alcohol can cause damage to the liver cells, which emit lipid peroxidation products and reactive oxygen species (ROS). Immediately, the latter breaks down lipids, proteins, and DNA, which can trigger necrosis and hepatocyte death [3]. ROS also contributes to the intensification of the inflammatory response, ultimately leading to the onset of fibrosis [20]. ROS can potentially induce the synthesis of pro-fibrotic mediators from inflammatory cells that have been infiltrated in the affected area. Significantly, ROS can directly engage with HSCs, serving as the primary agents responsible for the production of ECM during fibrosis [19]. The activation of dormant HSCs is thus believed to be caused by alterations in the cellular redox state.

On the other hand, to control deleterious oxidative stress, each cell has self-protective mechanisms to maintain cellular redox homeostasis. The study conducted by Yang et al. [21] showed that miR-200a/ nuclear erythroid factor-2 related factor-2 (Nrf2) axis deletion caused severe inflammation and fibrosis and that the activation of miR-200a/ Nrf2 is advantageous in reducing collagen deposition and preventing liver fibrosis. Since Nrf2 is the primary regulator of genes engaged in the cellular defence mechanism against oxidative stress, it is a target for managing oxidative stress-related diseases [22]. The cytosolic protein known as Kelch-like ECH-associated protein 1 (Keap1) is a crucial player in controlling the expression and activity of Nrf2. At rest, the Nrf2 protein is susceptible to ubiquitination and proteasomal degradation processes that are controlled by Keap1. Consequently, decreasing the cytoplasmic Keap1 can enhance the expression of Nrf2 [23]. Notably, short non-coding RNA known as miR-200a encourages the degradation of Keap1 mRNA, increasing the expression of Nrf2 [24].

### Inflammation in liver fibrosis

In the early stages of hepatic damage, inflammation is brought on by the first cell death to aid in the elimination of cell debris and to encourage liver regeneration, which guarantees the restoration of the architecture and function of the liver after injury [25, 26]. If the underlying condition is not treated and the stressors continue, they lead to chronic inflammation and progressive liver fibrosis [27]. Increasing evidence indicates that liver fibrosis progression is facilitated by synergistic interactions between inflammatory activities and oxidative stress [28]. According to Dutta et al. [29], when hepatic oxidative damage occurs, it triggers the activation of HSCs and the recruitment of inflammatory and immunological cells, such as KCs, to the injured area. In response to inflammation, activated KCs secrete inflammatory mediators such as platelet-activating factor (PAF), which attaches to its G-protein-coupled receptor in hepatocytes. As a result, nuclear factor kappa-B p65 (NF-κB p65) is activated and translocated to the nucleus, which in turn causes an upregulation of several pro-inflammatory cytokines like tumour necrosis factor-alpha (TNF-α) [30, 31]. Notably, PAF and TNF-α have a direct relationship where PAF can increase TNF-α production in human monocytes and macrophages and vice versa [32, 33].

### Necroptotic and fibrotic signalling pathways in liver fibrosis

Necroptosis is a kind of programmed cell death that primarily manifests as necrosis-like morphological features, such as inflammation and irregularities in membrane integrity. It relies on mixed lineage kinase domain-like (MLKL) and receptor-interacting serine-threonine kinases (RIPKs). The necroptotic pathway has recently been discovered to play a significant part in fibrosis pathophysiology. Numerous investigations established that ROS and TNF-α are prototypical triggers for kicking off the necroptosis cascade [34, 35]. Linking of pro-inflammatory TNF-α with its receptor arouses the downstream molecules (RIPK1, RIPK3, and MLKL). Additionally, activated MLKL oligomerizes and creates gaps in the phospholipids in the membrane, which causes an increase in osmotic pressure, the release of cell components, and, ultimately, the rupture of the plasma membrane [36]. The released intracellular components, including damage-associated molecular patterns such as HMGB1, intensify the inflammatory cascade by releasing additional TNF-α, hence bolstering necroptosis [37].

On top of that, the stimulation of the RIPK3/MLKL/HMGB1 axis promotes fibrosis, as HMGB1 upregulates the production of TGF-β1 and α-SMA while inhibiting the action of collagen-degrading metalloproteinase-2. Additionally, HMGB1 activates HSCs and demonstrates pro-fibrogenic effects by increasing the population of HSCs and the contents of the ECM or converting HSCs into myofibroblasts [38]. In turn, activated HSCs secrete cytokines, such as TGF-β1, which stimulate more quiescent HSCs [39]. It is important to note that the aberrant increase in ROS levels associated with miR-200a dysregulation leads to increased expression of HMGB1 and TGF-β1-mediated α-SMA in hepatic fibrosis [5].

### Hh cascade in liver fibrosis

The Hh cascade is one signalling system correlated to tissue patterning and embryonic development. Overly abundant deposition of ECM and epithelial-mesenchymal transition (EMT) are linked to Hh signalling activation. In contrast, the Hh signalling blockade inhibits the EMT and alleviates tissue fibrosis [40]. The patched-1 receptor (PTCH-1) binds to the smoothened transmembrane protein (SMO) in a normal adult liver, where Hh signalling is perceived as dormant and Hh ligands are absent [41, 42]. Fibrosis-related growth factors and cytokines, such as TGF-β1, induce HSCs to generate Hh ligands [3, 43]. The latter promotes the growth and survival of myofibroblasts and the surrounding Hh-responsive cells by binding to their PTCH-1 receptors [3]. This binding results in the release of SMO protein, which in turn stimulates the nuclear translocation of the glioma-associated oncogene analogue (Gli) transcription factors (Gli-1 and Gli-2) [44, 45]. The activation of numerous target genes involved in myofibroblast formation, including α-SMA and TGF-β1, is facilitated by Gli-2 galvanizing and Gli-1 functioning as a signal amplifier of Gli-2. In addition, TGF-β1 significantly stimulates the transcription of the Gli2 and Hh axis [44, 46].

### Pathogenic angiogenesis in liver fibrosis

Angiogenesis is a hypoxia and growth factor-dependent process that occurs at every stage of human development. It is common knowledge that inflammation and hypoxia are necessary for neovascularization in fibrosis affecting hepatic tissues [47]. Experimental studies showed that angiogenesis hastens the development of liver fibrosis [48, 49]. In particular, hypoxia is primarily caused by ECM build-up in the liver parenchyma as well as changes in the structure of liver LSECs where hypoxia initiates an intracellular pathway to activate the hypoxia-inducible factor-1alpha (HIF-1α) [47, 48]. Of note, the transcription of numerous genes is regulated by HIF-1α, including genes that play a role in angiogenesis. Importantly, VEFG stimulates LSECs and initiates angiogenesis in conjunction with the activation of HSCs [6, 47]. Several chemokines with a CXC motif are expressed by activated HSCs and can manipulate angiogenesis. Furthermore, hypoxia encourages inflammatory cell infiltration even more, aiding in the angiogenic and fibrotic processes [47, 50]. Thus, the severity of liver fibrosis is worsened by the interplay of hypoxia, angiogenesis, and inflammation, which are all interconnected and negatively impact each other.

## Pathogenic EXOs in liver fibrosis

EXOs play a crucial role in the pathophysiology of liver fibrosis by controlling HSCs activation and immune cell interaction [51]. The liver’s cellular components, including hepatocytes, HSCs, and LSECs, can secrete or act as target cells for EXOs [52]. The interaction between these cells through EXOs is crucial to initiate and proceed hepatic fibrosis [52, 53]. The phenotypic switch of quiescent stellate cells occurs when EXOs released by damaged hepatocytes internalize in HSCs. The activated HSCs release more EXOs that are rich in fibrotic contents, which can promote extra adjacent HSCs activation and fibrosis by different mechanisms [54, 55]. Sphingosine kinase 1 in LSEC-derived EXOs also enhances the migration and transformation of neighbouring HSCs into myofibroblasts [56]. Furthermore, studies have demonstrated that macrophage-derived exosomal miR-103-3p and miR-500 can stimulate HSCs activation and the process of liver fibrosis [57, 58]. In addition, exosomal dihydrofolate reductase secreted by activated HSCs can further promote M1 polarization in macrophages, which fastens liver fibrosis [59]. Two glycolysis-related proteins which are glucose transporter type 1 and pyruvate kinase isozyme type M2, are transported by HSC-derived EXOs, influencing the metabolic switch in non-parenchymal cells of the liver and playing a significant role in liver fibrosis [60]. Moreover, hepatic EXOs may activate toll-like receptor 3 in HSCs, which in turn stimulates gamma delta T cell IL-17 production and worsens liver fibrosis [61]. Collectively, hepatic damage stimulates the activation of monocytes and macrophages and generates a cascade of pro-inflammatory mediators that induce further inflammation and fibrosis via EXOs [62]. Thus, EXOs are essential mediators of intercellular communication that are involved in the pathogenesis of liver fibrosis via transporting cytokines, regulating immune responses, and affecting the signalling pathways associated with inflammation and fibrosis.

## Management of liver fibrosis

Liver fibrosis was previously assumed irreversible, but recent research has shown that even severe fibrosis can be reversed. Eliminating the stimulus or detrimental source of liver injury is the most efficient strategy to inhibit fibrosis. However, it could be years before meaningful improvement is seen, depending on the severity of the disease [63]. Among various efforts to alleviate liver fibrosis, several therapies have emerged to suppress ongoing liver injury, such as anti-inflammatory and anti-fibrotic therapies; where these current therapies can be classified into different categories: removing the stimulus or damaging cause, dwindling hepatic inflammation, regulating the immune response, inhibiting HSCs activation, and encouraging ECM degradation via modulating receptor-ligand interactions and intracellular signalling [64]. Although there is an increasing number of drugs being experimentally evaluated, there are currently no clinically licensed anti-fibrotic medicines. This may arise from the fact that many types of cells and pathways have been shown to contribute to liver fibrosis, demonstrating just how intricate the hepatic fibrosis mechanisms are [65]. Also, most currently available medications are directed towards only a single factor or target (e.g., inflammation, oxidative stress, or angiogenesis) rather than numerous targets. Over and above, there is a large discrepancy between the diseased conditions shown in animal models and those observed in patients, contributing to the lacklustre medication performance observed in clinical trials [66].

Recent advances in liver fibrosis treatment have opened up new avenues of hope, such as stem cell therapy, which has shown encouraging results in preclinical and clinical trials [67]. The capacity of MSCs, derived from various sources such as bone marrow, amniotic fluid, adipose tissues, dental pulp, and umbilical cord, to self-renew and differentiate into numerous cell types makes them an attractive option in regenerative medicine [68]. Early research demonstrated the MSCs’ ability to reach target tissues as a means of their therapeutic effectiveness. Afterwards, it became clear that the biological impacts were mediated by growth factors, cytokines, chemokines, and EVs generated by MSCs [69]. These biological mediators either maintain the ability of MSCs to self-renew, differentiate, and proliferate (autocrine functions) or modulate the immune system, inflammatory response, and apoptosis in neighbouring cells (paracrine functions) [70]. Notably, the primary element contributing to MSCs’ paracrine effects is EVs [69].

EVs are heterogeneous nanoparticles that are encircled by a phospholipid membrane and contain transmembrane proteins, cytosolic proteins, organelles, transcription factors, mRNAs, miRs, and a variety of signal transduction molecules. They are typically found in MSCs, tumour cells, fibroblasts, neurons, endothelial cells, and epithelial cells, where they function as versatile mediators between adjacent or distant cells in various physiological and pathological processes. EVs can be loosely categorized into three primary classes based on their mechanism of release and size: EXOs, microvesicles, and apoptotic bodies [70, 71].

**Fig. 2:**
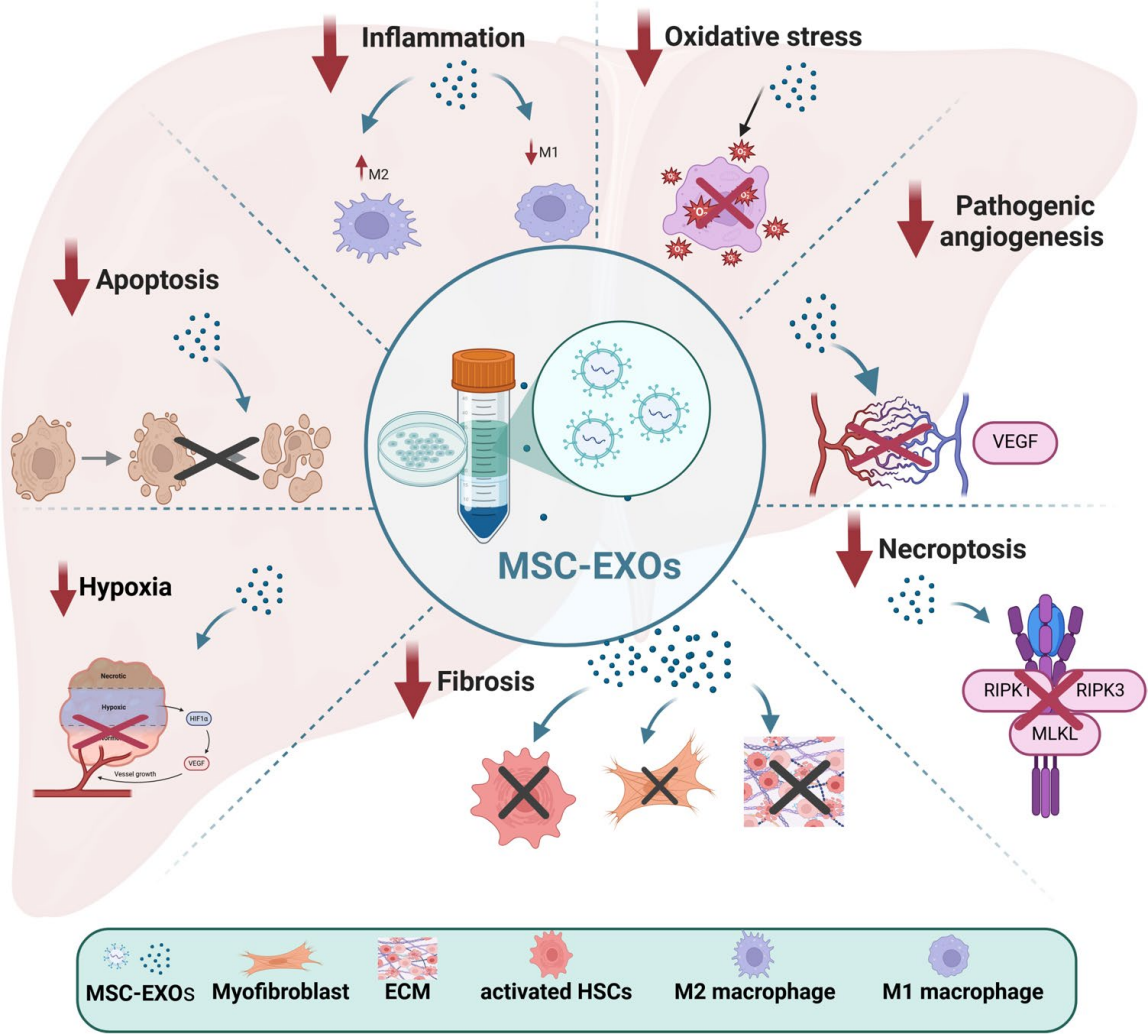
MSC-EXOs as a possible approach for management of hepatic fibrosis. The targeted cargo, such as proteins and nucleic acids, is delivered to injured liver tissues via EXOs, which then exercise their therapeutic effects through several mechanisms. ECM, extracellular matrix; HIF-1α, hypoxia-inducible factor-1alpha; HSCs, hepatic stellate cells; MLKL, mixed lineage kinase domain-like; MSC-EXOs, mesenchymal stem cell-derived exosomes; RIPKs, receptor-interacting serine-threonine kinases; VEGF, vascular endothelial growth factor (Created in *BioRender.com*)

It is well known that EXOs are tiny membrane vesicles with sizes ranging from 40 to 150 nm that are responsible for intercellular communication by facilitating the transfer of bioactive lipids, proteins, and RNAs [72]. As demonstrated in Fig. (2), previous reports suggested that the administration of MSC-EXOs has yielded beneficial outcomes in several liver fibrosis models due to their regenerative, immunomodulatory, anti-oxidant, anti-inflammatory, anti-apoptotic, and anti-fibrotic properties [5, 11, 73, 74]. For instance, human umbilical cord MSC-EXOs alleviated liver fibrosis through obstructing the TGF-β1/SMAD axis and impeding the harmful EMT [75]. Specifically, miR-148a released from human umbilical cord MSC-EXOs was shown to regulate intrahepatic macrophage via remodelling of pro-inflammatory macrophage phenotypes, controlling Kruppel-like factor 6/ signal transducer and activator of transcription 3 (STAT3) activity and, consequently, inhibiting liver fibrosis progression [73]. In addition, ohara et al. [76] stated that amnion-MSC-EXOs improved liver inflammation and fibrosis via suppressing the activation of HSCs and KCs. Recently, Chen et al. [74] proved that MSC-EXOs attenuated liver fibrosis in primary sclerosing cholangitis by inhibiting T helper 17 cells differentiation and the provoked inflammation. Interestingly, MSC-EXOs can target HSCs to prevent their activation and proliferation, thereby reducing the expression of collagen and other fibrosis-related genes [75, 77, 78]. In the same pattern, Tan et al. [79] revealed that Beclin1 supplied by human umbilical cord MSC-EXOs relieved liver fibrosis via reduction of HSCs glutathione peroxidase 4 and consequently activating their ferroptosis. Furthermore, Rong et al. [11] evidenced that rat bone marrow MSC-EXOs can mitigate carbon tetrachloride (CCl_4_)-induced liver fibrosis by endorsing hepatocyte regeneration and impeding HSCs activation via activation of Wnt/β-catenin pathway both in vivo and in vitro. Importantly, the application of MSC-EXOs possesses several advantages over their corresponding MSCs, including traversing biological barriers in addition to possessing a safer profile, lower immunogenicity, and a smaller and simpler structure than their parent cells, which in turn facilitates their production and storage [11]. Badillo et al. 2007; Jeong et al. 2011 and Wang et al. 2015b [80–82] have shown that EXOs may be capable of circumventing specific regulatory challenges that MSCs encounter, including immune rejection, entrapment in the lung microvasculature, and ectopic tumour growth. Accordingly, MSC-EXOs have gained a lot of interest as a promising treatment option for treating liver fibrosis in the last decades.

Notwithstanding these previous evident benefits, there are several challenges facing the clinical application of MSC-EXOs, including the low yield of MSC-EXOs in traditional culture media [83] and the substantial decrease in the therapeutic efficacy of secreted EXOs from MSC senescence after multiple in vitro generations. Importantly, the produced EXOs are not also homogeneous [84]. Furthermore, it is believed that native or unmodified EXOs have inadequate targeting characteristics at the injury site after intravenous administration, which might impede the therapeutic effect of MSC-EXOs in the injured area. Additionally, the degradation of EXOs in response to elevated oxidative stress in diseased conditions and the subsequent activation of cellular autophagy may contribute to their reduced efficacy [10, 85]. Collectively, many factors can affect EXOs composition, including the MSC’s culture condition, the isolation method, and their biological source [86–88]. Thus, it is vigorously advised to enhance their targeting and therapeutic effectiveness in addition to their produced amounts through different strategies to expand their clinical uses.

## Engineering approaches of EXOs

The engineering of MSC-EXOs is a process that involves augmenting the therapeutic efficacy and regenerative potentials of the MSC-EXOs as demonstrated in Table 1, leading to a more substantial targeting impact via transferring and supplying bioactive components (such as lipids, proteins, miRs,…etc.) to obtain high and unique performance [89]. These outcomes can be achieved by two methods; the first encompasses loading endogenous or exogenous cargo (nucleic acids, proteins, and drugs) into the lumen of EXOs, and the second one is achieved by surface modification of MSC-EXOs to target them to a particular type of tissue or cells [13, 84, 90].

**Table 1:** Examples of the therapeutic efficacy of engineered MSC-EXOs in hepatic fibrosis

No.	Title of study	Method	Outcomes	References
1	MiR-122 modification enhances the therapeutic efficacy of adipose tissue-derived mesenchymal stem cells against liver fibrosis	Excessive miR-122 expression: Transfection of MSCs with lentivirus encoding miR-223-3p.	Enhancing the anti-fibrotic effects of AMSCs by reducing HSCs proliferation and collagen maturation through EXOs-mediated miR-122 communication between donor AMSCs and host HSCs.	[94]
2	Exosomes derived from miR-181-5p-modified adipose-derived mesenchymal stem cells prevent liver fibrosis via autophagy activation	Overexpressed miR-181-5p: Transfection of MSCs with plasmids encoding miR-223-3p.	Activating autophagy and mitigating hepatic fibrosis caused by TGF-β1 through blocking the STAT3/Bcl-2/Beclin 1 pathway.	[95]
3	Therapeutic potential of exosome derived from hepatocyte growth factor-overexpressing adipose mesenchymal stem cells in TGFβ1-stimulated hepatic stellate cells	Lentiviral transfection of parental mouse adipose MSCs for obtaining HGF-loaded MSC-EXOs.	Alleviating liver fibrosis via inhibition of the Rho pathway.	[96]
4	Effects of mesenchymal stem cells-derived extracellular vesicles on inhibition of hepatic fibrosis by delivering miR-200a	Lentiviral transfection of parental amniotic-MSCs for obtaining miR-200a-loaded MSC-EXOs.	Conveying miR-200a to hepatocytes and upregulating its expression in hepatocytes as well as inhibiting the expression of ZEB1, the transcription of PIK3R3, and ultimately the process of hepatic fibrosis.	[97]
5	Mesenchymal stem cell-derived exosomal miR-26a induces ferroptosis, suppresses hepatic stellate cell activation, and ameliorates liver fibrosis by modulating SLC7A11	Overexpressed miR-26a: Transfection of MSCs with vector encoding miR-26a.	Inducing ferroptosis and alleviating hepatic fibrosis by modulating miR-26a/SLC7A11 axis.	[98]
6	Human umbilical cord perivascular cells-derived extracellular vesicles mediate the transfer of IGF-I to the liver and ameliorate hepatic fibrogenesis in mice	Overexpressed IGF-1 expression: Transfection of MSCs with lentivirus encoding IGF-1.	Reducing inflammatory events and hepatic fibrosis.	[99]
7	BMP7-loaded human umbilical cord mesenchymalstem cell-derived small extracellular vesiclesameliorate liver fibrosis by targeting activatedhepatic stellate cells	Lentiviral transfection of parental hucMSCs for obtaining BMP7-loaded MSC-sEVs.	Overexpressing the anti-fibrotic BMP7 gene in parental MSCs and enhancing the anti-fibrotic effects of MSC-sEVs by promoting aHSC phenotypic reversion and inhibiting its proliferation.	[100]
8	Mesenchymal stem cell-originated exosomal circDIDO1 suppresses hepatic stellate cells miR-141-3p/PTEN/AKT pathway in human liver fibrosis.	Excessive circDIDO1 expression: The plasmid was transiently transfected into MSC after the full-length circDIDO1 cloning into the pLC5-ciR vector.	Dwindling the activated HSCs by increasing PTEN to decrease AKT pathway through miR-143-3p.	[101]
9	Therapeutic targeting of STAT3 with small interference RNAs and antisense oligonucleotides embedded exosomes in liver fibrosis	siRNA and ASO were developed to specifically target STAT3 using electroporation as a loading method.	Enhancing STAT3 targeting efficacy, reducing both STAT3 levels and ECM deposition, enhancing liver functions, and further inhibiting liver fibrosis as compared to native EXOs.	[110]
10	Effective delivery of osteopontin small interference RNA using exosomes suppresses liver fibrosis via TGF-β1 signalling	OPN siRNA-electroporated EXOs were administered to rodents with CCl4-induced liver fibrosis.	Alleviating liver fibrosis and the deposition of ECM via inhibition of OPN/HMGB1/TGF-β1 axis.	[111]
11	Obeticholic acid-loaded exosomes attenuate liver fibrosis through dual targeting of the FXR signaling pathway and ECM remodeling	Obeticholic acid was loaded into MSC-EXOs using ultrasound.	Stimulating the FXR axis and thus promoting ECM breakdown by increasing MMP-13 levels and decreasing TIMP-1 levels, and further inhibiting liver fibrosis.	[115]
12	Luteolin-loaded exosomes derived from bone marrow mesenchymal stem cells: a promising therapy for liver fibrosis	LUT was loaded into MSC-EXOs using the sonication and the physical incubation method.	Enhancing of LUT uptake into hepatocytes with further inhibition of liver fibrosis using LUT-EXOs as compared to LUT suspension or blank EXOs	[117]
13	Huc-MSC-derived exosomes modified with the targeting peptide of aHSCs for liver fibrosis therapy	The HSTP1 peptide was fused with an exosomal-enriched membrane protein (Lamp2b) on the surface of EXOs	Enhancing the capacity of EXOs to reverse liver fibrosis, reducing collagen deposition, and modulating M2 macrophage polarization by inhibiting CCl2 secretion from activated HSCs	[123]

*aHSCs* activated hepatic stellate cells, *AKT* protein kinase B, *AMSCs *adipose tissue-derived mesenchymal stem cells, *ASO* antisense oligonucleotides, *Bcl2* proapoptotic proteins B-cell lymphoma-2, *BMP7* gene bone morphogenic protein 7, *CCL2* chemokine (C-C motif) ligand 2, *CCL4* carbon tetrachloride, *ECM* extracellular matrix, *FXR* farnesoid X receptor, *HGF* hepatocyte growth factor, *HMGB1* high mobility protein box1, *HucMSCs* human umbilical cord-derived mesenchymal stem cells, *IGF-1* insulin growth factor like-I, *Lamp2b* lysosomal associated membrane protein 2, *LUT* Luteolin, *miRs* micro-RNAs, *MMP-13* matrix metalloproteinase-13, *MSC-EXOs* mesenchymal stem cells-derived exosomes, *OPN* osteopontin, *PI3K *phosphoinositide 3-kinase, *PIK3R3* phosphoinositide-3-kinase regulatory subunit 3, *PTEN* phosphatase and tensin homolog, *sEVs* small extracellular vesicles, *siRNA* small interfering RNAs, *STAT* signal transducer and activator of transcription, *TIMP-1* tissue inhibitor metalloproteinase 1, *TGF-β1* transforming growth factor-beta 1, *ZEB1* zinc finger E-box binding homeobox 1

### Cargo loading into EXOs

#### Endogenous cargo loading (cell-based loading)

As the status of MSCs determines the properties of their derived EXOs [91], it is possible to genetically modify MSCs using different biological tools, such as plasmids or viral vectors. These methods allow the cells to produce endogenous compounds, which can then be encapsulated inside MSC-EXOs, utilising the cell’s biomolecular production mechanism [92, 93]. Thus, genetic engineering approaches of parental MSCs could be used to enhance the immunomodulatory, reparative, and regenerative activities of their derived EXOs in addition to the elimination of batch-to-batch heterogeneity [84].

The use of genetically engineered MSCs could affect EXOs efficacy through miRs overexpression. For instance, Lou et al. [94] used lentivirus to transfect miR-122 into MSCs and successfully isolated EXOs with overexpressed miR-122 to target HSCs in vitro, inhibiting their activation and amending fibrosis by downregulating insulin-like growth factor receptor 1, cyclin G, and prolyl-4-hydroxylase α1, which successfully prevented proliferation and collagen maturation of HSCs compared to native EXOs. Similarly, EXOs with overexpressed miR-181b from adipose MSCs were demonstrated to upsurge autophagy and diminish TGF-β1-induced hepatic fibrosis by inhibiting STAT3/proapoptotic proteins B-cell lymphoma-2/Beclin 1 axis in mouse hepatic stellate (HST-T6 ) cells in vitro and CCl4-induced hepatic fibrosis in vivo [95]. Furthermore, Wang et al. [96] verified that EXOs produced from transfecting hepatocyte growth factor into adipose MSCs ameliorated liver fibrosis by inhibiting the Rho pathway and consequently decreasing the generation of collagen. Also, Xu et al. [97] proved that human amniotic MSCs-derived EVs loaded with miR-200a increased the expression of miR-200a in hepatocytes, which in turn inhibited the intracellular zinc finger E-box binding homeobox 1/ phosphoinositide-3-kinase regulatory subunit 3 axis and achieved significant anti-fibrotic effects. Similarly, miR-26a-enriched MSC-EXOs provoked ferroptosis of HSCs to ameliorate liver fibrosis by regulating SLC7A11 [98].

Besides miRs, the piggybacking of endogenous proteins has improved the therapeutic efficacy of MSC-EXOs in various diseases. Fiore et al. [99]used an adenoviral transfection system to transfect human insulin growth factor like-I (IGF-I) into human umbilical cord perivascular cells and found that IGF-I-MSC-EVs exhibited more anti-fibrotic effects in thioacetamide-induced liver fibrosis than EVs-depleted conditioned medium. Over and above, the therapeutic effect of morphogenic protein 7-loaded human umbilical cord-MSC-EXOs was significantly more substantial than that of blank or negatively controlled-transfected EXOs in reversal of activated HSCs and inhibition of liver fibrosis [100]. Also, human bone marrow MSCs transfected with circDIDO1 (a circular RNA derived from the DIDO1 gene) released EXOs, which  suppressed HSCs activation and liver fibrosis through the miR-141-3p/phosphatase and tensin homolog / protein kinase B axis [101].

#### Exogenous cargo loading (non-cell-based EXOs loading)

Exogenous or direct cargo loading deposits a therapeutic payload into EXOs after isolation. Sonication, electroporation, and a specific buffer agent can be used to load EXOs with various substances, including proteins, hydrophobic chemical-derived natural products, anticancer medications, small interfering RNAs (siRNA), and transfection reagents. These loading methods can be categorized as passive and active loading, where both may be integrated to enhance loading efficiency [102]. Passive loading entails the diffusion of therapeutic cargo into EXOs, while active loading involves the disruption of EXOs membranes via electroporation or sonication to facilitate cargo entry. Upon completion of the process, the membrane is retrieved by incubation at room temperature or 37 °C [103].

Most exogenous cargo is currently loaded into EXOs using electroporation [104]. This approach relies on an external electric field to create tiny repairable pores in the phospholipid bilayer. These pores then enable the loading of various small compounds into EXOs by applying an electric field force. Through the use of electroporation, Liang et al. [105] were able to incorporate exogenous anticancer medication norcantharidin into MSC-EXOs. Because of EXOs’ inherent liver-targeting capabilities, these modified EXOs were utilised to treat animal models of liver cancer. In other experimental models of colon and breast cancer [106, 107], anticancer medicines like doxorubicin could be efficiently delivered into MSC-EXOs using electroporation, where the tumour growth rates were dramatically reduced. On top of that, MSC-EXOs can be loaded with exogenous compounds by coincubation, sonication, and dialysis [84, 108]. For example, researchers co-incubated MSC-EXOs with siRNA of phosphatase and tensin homologs to increase the safety and efficacy of MSC-EXOs as a treatment for spinal cord injury with an improvement of axonal regeneration and restoration of motor function in rats [109].

The use of siRNAs and miRs has many benefits for targeting liver fibrosis. Tang et al. [110] demonstrated that MSC-EXOs loading with antisense oligonucleotide (EXOs-ASO) and siRNA (EXOs-siRNA) targeting STAT3 via electroporation reduced both STAT3 levels and ECM deposition as well as enhanced liver functions and inhibited fibrosis in an experimental model of CCl_4_-induced liver fibrosis. Similarly, Tang et al. [111] electroporated MSC-EXOs with siRNA-targeting osteopontin (siRNA-OPN) where engineered EXOs with siRNA-OPN showed superior efficacy regarding the deposition of ECM and amelioration of hepatic fibrosis via inhibition of OPN/HMGB1/TGF-β1 axis, as compared to naked siRNA-OPN. MSC-EXOs treated with galectin-9 siRNA also inhibited macrophage polarization, downregulated regulatory T cells, and ameliorated pancreatic ductal adenocarcinoma [112]. Greco et al. [113] electroporated MSC-EXOs with polo-like kinase 1(PLK-1) siRNA to silence PLK-1 in bladder cancer cells. In addition, Shojaei et al. [114] used electroporation to package miR-381-3p mimic into MSC-EXOs and deliver it to triple-negative breast cancer cells to inhibit their migration. Thus, the previously mentioned outcomes emphasize the feasibility of applying these approaches in liver fibrosis.

Indeed, mounting various medicines in MSC-EXOs via an exogenous loading strategy is also an approach that has been studied in liver diseases. Azizsoltani et al. [115] loaded obeticholic acid into human wharton’s jelly MSC-EXOs using water bath ultrasound to activate the farnesoid X receptor axis, upregulate matrix metalloproteinase-13 and downregulate tissue inhibitor of metalloproteinase-1, thus improving the degradation of ECM, and alleviating hepatic fibrosis. Fang et al. [116] used quercetin and vitamin A-loaded adipose MSC-EXOs to effectively treat acute liver injury in mice where quercetin improved the therapeutic efficacy of the EXOs, while vitamin A augmented their targeting to the liver. Importantly, quercetin- and vitamin A-loaded MSC-EXOs mitigated the rapid senescence-like response triggered by acute liver injury. Furthermore, according to Ashour et al. [117], luteolin (LUT)-, a plant flavonoid, loaded EXOs revealed better anti-fibrotic potential than LUT-suspension. This finding could be explained by the fact that blank EXOs have biological activity, which could enhance the drug’s efficacy. Furthermore, EXOs could help LUT penetrate liver cells, allowing the drug to exert its effect for a prolonged duration.

#### Surface modification of EXOs

Previous researches demonstrated that surface engineering of EXOs could be used to alter the homing peptides or ligands on EXOs` surface, giving them a longer half-life in systemic circulation as well as enhancing their targeting ability and therapeutic efficacy [72, 118, 119]. It is possible to classify the engineering modifications made to the surface of MSC-EXOs as either indirect or direct ones. Through genetic engineering, functional peptides or proteins can be expressed on the membrane of parental cells, resulting in EXOs with similar membrane proteins. On the other hand, isolated EXOs can be directly modified by manipulating functional groups on their surface through chemical coupling reactions, ligand-receptor interactions, multivalent electrostatic interactions, and lipid fusion [120, 121]. For example, lysosomal associated membrane protein 2 (Lamp2b) protein is the most frequently utilised site for fusing with targeting moieties for adhesion [122]. Through genetic engineering technology, HSTP1 peptide can be fused with Lamp2b and displayed on the surface of EXOs (HSTP1-EXOs). Lin et al. [123] have verified that HSTP1-EXOs could specifically target the HSCs region following intravenous injection and improve the therapeutic efficacy against hepatic fibrosis in an in-vivo investigation. Also, glycosylated sequence GNSTM conjugated with lamp2b augmented the expression of Lamp2b fusion proteins in cells and EXOs, thus preventing the degradation of lamp2b protein-linked targeting peptides and enhancing the efficacy of EXOs-linked targeting peptides [124]. One way to improve the hepatic targeting of MSC-EXOs is to modify its surface with cationized pullulan (a biomaterial with a strong affinity for the liver) using multivalent electrostatic interaction. Compared to unmodified EXOs, pullulan-modified ones accumulated more in the liver with acute damage utilising concanavalin in mice [125]. EVs could be also used with their targeting ligands conjugated to polyethylene glycol (PEG) with specific binding to epidermal growth factor receptor-overexpressing tumour cells where these EVs afforded improved cargo delivery and prolonged systemic circulation times [126]. Another study by Hwang et al. [127] indicated that anionic EXOs designed by conjugating EXOs surface proteins with near-infrared fluorophores exhibited high liver uptake with the hepatobiliary route as a main route of EXOs excretion. It has also been discovered that altering the arginine-rich cell-penetrating peptide on the surface of EXOs might enhance cellular uptake of EXOs by inducing macropinocytosis [128].

### Scaling-up of MSC-EXOs

Scaling up MSC-EXOs is a process that increases the produced amounts of EXOs while preserving their quality and function to fulfil their therapeutic and clinical application requirements. Scaling up can be achieved in two ways. One relies on genetic engineering to alter the EXOs’ biogenesis and their release mechanism, while the second depends on preconditioning the parental MSCs through optimising culture conditions, changing culture methods, or amending the medium with various exogenous stimulatory factors [129, 130].

Utilising genetic manipulation to overexpress the desired miRs and proteins in MSC-EXOs can improve the therapeutic effects and simultaneously enhance the production of EXOs [131]. Hassan et al. [132] also demonstrated that three-dimensional (3D) cell culture platforms, like cell spheres and bioreactors, could offer a vast surface area for cell growth, enabling the proliferation of a large number of cells densely, leading to a high yield of EXOs. For example, EXOs derived from 3D cultures of umbilical cord MSCs in a hollow-fibre bioreactor exhibit further improvement in osteochondral regeneration efficacy and a higher yield than those produced from conventional 2D tissue culture flasks [133]. Furthermore, the anti-fibrotic efficacy of EXOs derived from human embryonic stem cells was significantly higher in 3D spheroid culture than 2D monolayer culture. This could be achieved through upregulating miR-6766-3p, which inhibited the TGF-β1/SMAD axis and, in turn, prevented the activation of HSCs and liver fibrosis [134].

Moreover, multiple studies have shown that through MSCs preconditioning under hypoxic environments, MSCs release an increased amount of EXOs, which in turn increase their therapeutic potential. Obviously, hypoxia-treated MSC-EXOs ameliorated myocardial infarction experimentally compared to untreated MSC-EXO, which could be related to enhanced vascular density, decreased myocardial apoptosis, and reduced fibrosis [135]. Furthermore, adding rupatadine, an anti-histaminic medicine, significantly improved the in vivo homing and the therapeutic efficacy of MSC-EXOs in liver fibrosis in rats through creating a more favourable environment for EXOs action together with increasing miR-200a level and inhibiting oxidative stress, inflammatory, necroptotic, and Hh pathways [5]. In the same context, Wei et al. [136] showed that MSC-EXOs combined with glycyrrhetinic acid, a triterpenoid saponin found in liquorice root and rhizome extracts, enhanced the therapeutic potential of MSC-EXOs in liver ischemia/reperfusion injury with a significant increase in the expression of anti-inflammatory proteins and restoration of the dysregulated inflammation and oxidative stress compared to MSC-EXOs alone.

### Clinical research progress in the application of MSCs and their derived EXOs in liver fibrosis

MSCs have been investigated in several clinical trials as a potential treatment for liver fibrosis. For example, Kharaziha et al. [137] demonstrated that autologous bone marrow MSCs administration could considerably enhance liver function in patients with hepatic fibrosis. Moreover, allogeneic MSCs transplantation revealed improvements in serum albumin and a marked decrease in serum alanine aminotransferase and total bilirubin levels in 26 patients with autoimmune cirrhosis [138]. Furthermore, MSCs were used in conjunction with drugs to treat liver disease [139] where patients with compensated cirrhosis who were given an intraportal infusion of autologous MSCs along with pioglitazone revealed stable status, with no significant adverse effects or deterioration. In this trial, there was a temporary improvement in the end-stage liver disease scores 3 months post-infusion in patients, which returned to baseline levels after one year.

Afterwards, numerous clinical trials have begun to evaluate the therapeutic efficacy of MSC-EXOs in various diseases, including respiratory distress syndrome, renal disease, graft-versus-host disease, osteoarthritis, acne scars, and stroke [140–144]. Nonetheless, confirming the therapeutic effectiveness of MSC-EXOs upon clinical applications in various diseases encourages their clinical investigation in hepatic fibrosis. However, standardized protocols regarding their therapeutic dosage, administration route, and the origin of parent MSCs should be carefully assessed in addition to various drug-related factors [145]. Moreover, the stability and safety of MSC-EXOs, in combination with various pharmacological treatments, should be thoroughly investigated in detailed and mechanistic experimental studies before being applied clinically. Utilising regenerative medicine and pharmacological approaches together, we could pave the way for safer and more effective treatments for people suffering from liver fibrosis.

### Challenges and future perspectives of MSC-EXOs application in liver fibrosis

The resolution of current challenges in the application of MSC-EXOs could enhance their clinical translation. One potential limitation is MSC-EXOs heterogeneity that results from various cell passages and culture conditions where isolation techniques for MSC-EXOs need to be standardised to overcome this challenge. Obviously, to reduce variability in EXOs size, content, and function, researchers should use consistent procedures for separating EXOs. In addition, MSCs and their EXOs should be more uniformly produced using standardized culture conditions [140, 146]. Preventing MSCs’ senescence after multiple passages and keeping MSC-EXOs viable for an extended period are also other challenges that should be investigated. Lyophilizing MSC-EXOs or adding stabilizing chemicals like sugars or PEG could help EXOs last more extended periods, as demonstrated by multiple research groups [147–149].

Yet another difficulty that faces MSC-EXOs application is their rapid elimination from the body after systemic administration, which could affect their ability to provide long-term therapeutic benefits [150]. This limitation could be improved by utilising engineered EXOs with enhanced targeting capabilities and resistance to degradation, as stated herein, in addition to combining their use with pharmacological agents. Importantly, developing advanced animal models in preclinical studies is crucial for bridging the gap between preclinical and clinical research. For example, utilising humanized and genetically engineered systems could more precisely emulate human physiological conditions, especially in intricate disorders like liver fibrosis [151, 152]. These models could facilitate an enhanced comprehension of EXOs’ safety profile and biodistribution in addition to the identification of dependable biomarkers for assessing EXOs’ effectiveness and disease progression. Collectively, the use of these advanced preclinical models could facilitate the clinical translation and mitigate the risk of failure in human trials.

## Conclusion

MSC-EXOs are one of the promising therapies that have been investigated for the management of liver fibrosis. However, their clinical application is limited by poor targeting and stability after in vivo transplantation. Engineering technique is one of the recent approaches that have been used to enhance the therapeutic efficacy of MSC-EXOs and their molecular targeting properties. This approach manipulates EXOs components by modifying their parent cells or directly changing the derived EXOs by specific therapeutic molecule loading or surface modification. As a consequence, this technique can modulate the activation or inhibition of the main signalling pathways that are involved in the progression of liver fibrosis. Despite their encouraging outcomes, engineering approaches necessitate extra researches and trials for optimising the techniques used, including physical loading, chemical modification, or transfection methods. Therefore, additional studies and trials are still needed to overcome the challenges and difficulties to allow successful clinical applications of EXOs in liver fibrosis.

## Data Availability

Data sharing is not applicable to this article as no data set was generated or analysed during the current study.

## References

[1] Nan Y, Su H, Lian X, Wu J, Liu S, Chen P, et al. Pathogenesis of liver fibrosis and its TCM therapeutic perspectives. Evid Based Complement Altern Med. 2022;(1):5325431. 10.1155/2022/5325431 PMC9071861 35529927

[2] Elbaset MA, Mohamed BMSA, Hessin A, Abd El-Rahman SS, Esatbeyoglu T, Afifi SM, et al. Nrf2/HO‐1, NF‐κB and PI3K/Akt signalling pathways decipher the therapeutic mechanism of pitavastatin in early phase liver fibrosis in rats. J Cell Mol Med. 2024;28:e18116. 38214394 10.1111/jcmm.18116 PMC10844702

[3] El-Agroudy NN, El-Naga RN, El-Razeq RA, El-Demerdash E. Forskolin, a hedgehog signalling inhibitor, attenuates carbon tetrachloride-induced liver fibrosis in rats. Br J Pharmacol. 2016;173:3248–60. 27590029 10.1111/bph.13611 PMC5071558

[4] Roehlen N, Crouchet E, Baumert TF. Liver fibrosis: mechanistic concepts and therapeutic perspectives. Cells. 2020;9:1–43. 10.3390/cells9040875 PMC7226751 32260126

[5] Didamoony MA, Atwa AM, Ahmed LA. Modulatory effect of rupatadine on mesenchymal stem cell-derived exosomes in hepatic fibrosis in rats: a potential role for miR-200a. Life Sci. 2023;324: 121710. 37084952 10.1016/j.lfs.2023.121710

[6] Yang X, Wang Z, Kai J, Wang F, Jia Y, Wang S, et al. Curcumol attenuates liver sinusoidal endothelial cell angiogenesis via regulating Glis-PROX1-HIF-1α in liver fibrosis. Cell Prolif. 2020;53:e12762. 32119185 10.1111/cpr.12762 PMC7106966

[7] Ding Y, Luo Q, Que H, Wang N, Gong P, Gu J. Mesenchymal stem cell-derived exosomes: a promising therapeutic agent for the treatment of liver diseases. Int J Mol Sci. 2022;23:10972. 36142881 10.3390/ijms231810972 PMC9502508

[8] Gowen A, Shahjin F, Chand S, Odegaard KE, Yelamanchili SV. Mesenchymal stem cell-derived extracellular vesicles: challenges in clinical applications. Front cell Dev Biol. 2020;8:1–10. 32226787 10.3389/fcell.2020.00149 PMC7080981

[9] Liu Q, Li S, Dupuy A, Le Mai H, Sailliet N, Logé C et al. Exosomes as new biomarkers and drug delivery tools for the Prevention and Treatment of various diseases: current perspectives. Int J Mol Sci. 2021;22(15):7763. 10.3390/ijms22157763 PMC8346134 34360530

[10] Didamoony MA, Soubh AA, Atwa AM, Ahmed LA. Innovative preconditioning strategies for improving the therapeutic efficacy of extracellular vesicles derived from mesenchymal stem cells in gastrointestinal diseases. Inflammopharmacology. 2023;1:1–21. 10.1007/s10787-023-01350-6 PMC10692273 37874430

[11] Rong X, Liu J, Yao X, Jiang T, Wang Y, Xie F. Human bone marrow mesenchymal stem cells-derived exosomes alleviate liver fibrosis through the Wnt/β-catenin pathway. Stem Cell Res Ther. 2019;10:1–11. 30885249 10.1186/s13287-019-1204-2 PMC6421647

[12] Wei W, Ao Q, Wang X, Cao Y, Liu Y, Zheng SG, et al. Mesenchymal stem cell-derived exosomes: a promising biological tool in nanomedicine. Front Pharmacol. 2021;11: 590470. 33716723 10.3389/fphar.2020.590470 PMC7944140

[13] Lu Y, Mai Z, Cui L, Zhao X. Engineering exosomes and biomaterial-assisted exosomes as therapeutic carriers for bone regeneration. Stem Cell Res Ther. 2023;14:1–19. 36978165 10.1186/s13287-023-03275-x PMC10053084

[14] Yin T, Liu Y, Ji W, Zhuang J, Chen X, Gong B, et al. Engineered mesenchymal stem cell-derived extracellular vesicles: a state-of-the-art multifunctional weapon against Alzheimer’s disease. Theranostics. 2023;13:1264–85. 36923533 10.7150/thno.81860 PMC10008732

[15] Khanam A, Saleeb PG, Kottilil S. Pathophysiology and treatment options for hepatic fibrosis. Can It Be Completely Cured? Cells 2021. 2021;10:10:1097. 34064375 10.3390/cells10051097 PMC8147843

[16] Matsuda M, Seki E. The liver fibrosis niche: novel insights into the interplay between fibrosis-composing mesenchymal cells, immune cells, endothelial cells, and extracellular matrix. Food Chem Toxicol. 2020;143:111556. 32640349 10.1016/j.fct.2020.111556 PMC7484466

[17] Wynn TA, Ramalingam TR. Mechanisms of fibrosis: therapeutic translation for fibrotic disease. Nat Med. 2012;18:1028–40. 22772564 10.1038/nm.2807 PMC3405917

[18] Tsukada S, Parsons CJ, Rippe RA. Mechanisms of liver fibrosis. Clin Chim Acta. 2006;364:33–60. 16139830 10.1016/j.cca.2005.06.014

[19] Sanchez-Valle V, Chavez-Tapia C, Uribe N, Mendez-Sanchez M. Role of oxidative stress and molecular changes in liver fibrosis: a review. Curr Med Chem. 2012;19:4850–60. 22709007 10.2174/092986712803341520

[20] Li S, Hong M, Tan H-Y, Wang N, Feng Y. Insights into the role and interdependence of oxidative stress and inflammation in liver diseases. Oxid Med Cell Longev. 2016;2016;4234061. 10.1155/2016/4234061 PMC5192343 28070230

[21] Yang JJ, Tao H, Hu W, Liu LP, Shi KH, Deng ZY, et al. MicroRNA-200a controls Nrf2 activation by target Keap1 in hepatic stellate cell proliferation and fibrosis. Cell Signal. 2014;26:2381–9. 25049078 10.1016/j.cellsig.2014.07.016

[22] Ngo V, Duennwald ML. Nrf2 and oxidative stress: a general overview of mechanisms and implications in human disease. Antioxidants. 2022;11(12):2345. 10.3390/antiox11122345 PMC9774434 36552553

[23] Didamoony MA, Atwa AM, Abd El-Haleim EA, Ahmed LA. Bromelain ameliorates D-galactosamine-induced acute liver injury: role of SIRT1/LKB1/AMPK, GSK3β/Nrf2 and NF-κB p65/TNF-α/caspase-8, -9 signalling pathways. J Pharm Pharmacol. 2022;74:1765–75. 36227279 10.1093/jpp/rgac071

[24] Zhao XJ, Yu HW, Yang YZ, Wu WY, Chen TY, Jia KK, et al. Polydatin prevents fructose-induced liver inflammation and lipid deposition through increasing miR-200a to regulate Keap1/Nrf2 pathway. Redox Biol. 2018;18:124–37. 30014902 10.1016/j.redox.2018.07.002 PMC6068203

[25] Goodman ZD. Grading and staging systems for inflammation and fibrosis in chronic liver diseases. J Hepatol. 2007;47:598–607. 17692984 10.1016/j.jhep.2007.07.006

[26] Ganz M, Bukong TN, Csak T, Saha B, Park JK, Ambade A, et al. Progression of non-alcoholic steatosis to steatohepatitis and fibrosis parallels cumulative accumulation of danger signals that promote inflammation and liver tumors in a high fat-cholesterol-sugar diet model in mice. J Transl Med. 2015;13:193. 10.1186/s12967-015-0552-7 PMC4467677 26077675

[27] Czaja AJ. Hepatic inflammation and progressive liver fibrosis in chronic liver disease. World J Gastroenterol. 2014;20:2515–32. 24627588 10.3748/wjg.v20.i10.2515 PMC3949261

[28] Sharma N, Sistla R, Andugulapati SB. Yohimbine ameliorates liver inflammation and fibrosis by regulating oxidative stress and Wnt/β-catenin pathway. Phytomedicine. 2024;123: 155182. 37952411 10.1016/j.phymed.2023.155182

[29] Dutta S, Chakraborty AK, Dey P, Kar P, Guha P, Sen S, et al. Amelioration of CCl4 induced liver injury in Swiss albino mice by antioxidant rich leaf extract of Croton bonplandianus Baill. PLoS ONE. 2018;13: e0196411. 29709010 10.1371/journal.pone.0196411 PMC5927454

[30] Karantonis HC, Gribilas G, Stamoulis I, Giaginis C, Spiliopoulou C, Kouraklis G, et al. Platelet-activating factor involvement in thioacetamide-induced experimental liver fibrosis and cirrhosis. Dig Dis Sci. 2010;55:276–84. 19242794 10.1007/s10620-009-0745-0

[31] Borthakur A, Bhattacharyya S, Alrefai WA, Tobacman JK, Ramaswamy K, Dudeja PK. Platelet-activating factor-induced NF-κB activation and IL-8 production in intestinal epithelial cells are Bcl10-dependent. Inflamm Bowel Dis. 2010;16:593–603. 19714753 10.1002/ibd.21092 PMC3740729

[32] Dubois C, Bissonnette E, Rola-Pleszczynski M. Platelet-activating factor (PAF) enhances tumor necrosis factor production by alveolar macrophages. Prevention by PAF receptor antagonists and lipoxygenase inhibitors. J Immunol. 1989;143:964–70. 2545780

[33] Ruis NM, Rose JK, Valone FH. Tumor necrosis factor release by human monocytes stimulated with platelet-activating factor. Lipids. 1991;26:1060–4. 1668105 10.1007/BF02536502

[34] Hassanein EHM, Ibrahim IM, Abd El-Maksoud MS, Abd El-Aziz MK, Abd-alhameed EK, Althagafy HS. Targeting necroptosis in fibrosis. Mol Biol Rep. 2023;50(12):10471–84. 10.1007/s11033-023-08857-9 PMC10676318 37910384

[35] Fulda S. Regulation of necroptosis signaling and cell death by reactive oxygen species. Biol Chem. 2016;397:657–60. 26918269 10.1515/hsz-2016-0102

[36] Zhang X, Matsuda M, Yaegashi N, Nabe T, Kitatani K. Regulation of Necroptosis by phospholipids and sphingolipids. Cells. 2020;9:9. 10.3390/cells9030627 PMC7140401 32151027

[37] Zhang C, Dong H, Chen F, Wang Y, Ma J, Wang G. The HMGB1-RAGE/TLR-TNF-α signaling pathway may contribute to kidney injury induced by hypoxia. Exp Ther Med. 2019;17:17–26. 30651760 10.3892/etm.2018.6932 PMC6307518

[38] Li LC, Gao J, Li J. Emerging role of HMGB1 in fibrotic diseases. J Cell Mol Med. 2014;18:2331–9. 25284457 10.1111/jcmm.12419 PMC4302638

[39] Pang Q, Jin H, Wang Y, Dai M, Liu S, Tan Y, et al. Depletion of serotonin relieves concanavalin A-induced liver fibrosis in mice by inhibiting inflammation, oxidative stress, and TGF-β1/Smads signaling pathway. Toxicol Lett. 2021;340:123–32. 33429011 10.1016/j.toxlet.2021.01.010

[40] Hu L, Lin X, Lu H, Chen B, Bai Y. An overview of hedgehog signaling in fibrosis. Mol Pharmacol. 2015;87:174–82. 25395043 10.1124/mol.114.095141

[41] Omenetti A, Choi S, Michelotti G, Diehl AM. Hedgehog signaling in the liver. J Hepatol. 2011;54:366–73. 21093090 10.1016/j.jhep.2010.10.003 PMC3053023

[42] Chen J, Yang W, Dong T, Chen H, Zhang J, Xu G, et al. Quercetin ameliorates liver fibrosis in Wilson disease and EMT involving suppression of the hedgehog signaling pathway. Arab J Chem. 2024;17:105487.

[43] Mansour SM, El-Abhar HS, Soubh AA. MiR-200a inversely correlates with hedgehog and TGF-β canonical/non-canonical trajectories to orchestrate the anti-fibrotic effect of Tadalafil in a bleomycin-induced pulmonary fibrosis model. Inflammopharmacology. 2021;29:167–82. 32914382 10.1007/s10787-020-00748-w

[44] Jiayuan S, Junyan Y, Xiangzhen W, Zuping L, Jian N, Baowei H, et al. Gant61 ameliorates CCl4-induced liver fibrosis by inhibition of hedgehog signaling activity. Toxicol Appl Pharmacol. 2020;387: 114853. 31816328 10.1016/j.taap.2019.114853

[45] Yang X, Ji W, Zhou Z, Wang J, Cui Z, Pan X, et al. Dendrobium officinale polysaccharide regulated hepatic stellate cells activation and liver fibrosis by inhibiting the SMO/Gli 1 pathway. J Funct Foods. 2024;112: 105960.

[46] Kramann R. Hedgehog gli signalling in kidney fibrosis. Nephrol Dial Transpl. 2016;31:1989–95. 10.1093/ndt/gfw102 27229466

[47] Zadorozhna M, Di Gioia S, Conese M, Mangieri D. Neovascularization is a key feature of liver fibrosis progression: anti-angiogenesis as an innovative way of liver fibrosis treatment. Mol Biol Rep. 2020;47:2279–88. 32040707 10.1007/s11033-020-05290-0

[48] Melaibari M, Alkreathy HM, Esmat A, Rajeh NA, Shaik RA, Alghamdi AA, et al. Anti-fibrotic efficacy of apigenin in a mice Model of Carbon Tetrachloride-Induced hepatic fibrosis by modulation of oxidative stress, inflammation, and Fibrogenesis: a preclinical study. Biomedicines. 2023;11:11. 10.3390/biomedicines11051342 PMC10216039 37239014

[49] Didamoony MA, Atwa AM, Ahmed LA. A novel mechanistic approach for the anti-fibrotic potential of rupatadine in rat liver via amendment of PAF/NF-ĸB p65/TGF-β1 and hedgehog/HIF-1α/VEGF trajectories. Inflammopharmacology. 2023;31:845–58. 36811777 10.1007/s10787-023-01147-7 PMC10140091

[50] Feng J, Wang C, Liu T, Li J, Wu L, Yu Q, et al. Procyanidin B2 inhibits the activation of hepatic stellate cells and angiogenesis via the hedgehog pathway during liver fibrosis. J Cell Mol Med. 2019;23:6479–93. 31328391 10.1111/jcmm.14543 PMC6714206

[51] Liu Y, Zheng Y, Yang Y, Liu K, Wu J, Gao P, et al. Exosomes in liver fibrosis: the role of modulating hepatic stellate cells and immune cells, and prospects for clinical applications. Front Immunol. 2023;14:1–13. 10.3389/fimmu.2023.1133297 PMC10067730 37020547

[52] Wang C, Liu J, Yan Y, Tan Y. Role of exosomes in chronic liver disease development and their potential clinical applications. J Immunol Res. 2022;2022:1695802. 35571570 10.1155/2022/1695802 PMC9106457

[53] Gao J, Wei B, de Assuncao TM, Liu Z, Hu X, Ibrahim S, et al. Hepatic stellate cell autophagy inhibits extracellular vesicle release to attenuate liver fibrosis. J Hepatol. 2020;73:1144–54. 32389810 10.1016/j.jhep.2020.04.044 PMC7572579

[54] Higashi T, Friedman SL, Hoshida Y. Hepatic stellate cells as key target in liver fibrosis. Adv Drug Deliv Rev. 2017;121:27–42. 28506744 10.1016/j.addr.2017.05.007 PMC5682243

[55] Chen L, Chen R, Kemper S, Charrier A, Brigstock DR. Suppression of fibrogenic signaling in hepatic stellate cells by Twist1-dependent microRNA-214 expression: role of exosomes in horizontal transfer of Twist1. Am J Physiol Gastrointest Liver Physiol. 2015;309:G491-499. 26229009 10.1152/ajpgi.00140.2015 PMC4572411

[56] Wang R, Ding Q, Yaqoob U, De Assuncao TDM, Verma VK, Hirsova P, et al. Exosome adherence and internalization by hepatic stellate cells triggers sphingosine 1-Phosphate-dependent Migration. J Biol Chem. 2015;290:30684–96. 26534962 10.1074/jbc.M115.671735 PMC4692200

[57] Chen L, Huang Y, Duan Z, Huang P, Yao H, Zhou Y, et al. Exosomal miR-500 derived from lipopolysaccharide-treated macrophage accelerates liver fibrosis by suppressing MFN2. Front cell Dev Biol. 2021;9:716209. 10.3389/fcell.2021.716209 PMC8525629 34676206

[58] Chen L, Yao X, Yao H, Ji Q, Ding G, Liu X. Exosomal mir-103-3p from LPS-activated THP-1 macrophage contributes to the activation of hepatic stellate cells. FASEB J. 2020;34:5178–92. 32061112 10.1096/fj.201902307RRR

[59] Peng Y, Li Z, Chen S, Zhou J. DHFR silence alleviated the development of liver fibrosis by affecting the crosstalk between hepatic stellate cells and macrophages. J Cell Mol Med. 2021;25:10049–60. 34626074 10.1111/jcmm.16935 PMC8572769

[60] Wan L, Xia T, Du Y, Liu J, Xie Y, Zhang Y, et al. Exosomes from activated hepatic stellate cells contain GLUT1 and PKM2: a role for exosomes in metabolic switch of liver nonparenchymal cells. FASEB J. 2019;33:8530–42. 30970216 10.1096/fj.201802675R

[61] Seo W, Eun HS, Kim SY, Yi HS, Lee YS, Park SH, et al. Exosome-mediated activation of toll-like receptor 3 in stellate cells stimulates interleukin-17 production by γδ T cells in liver fibrosis. Hepatology. 2016;64:616–31. 27178735 10.1002/hep.28644

[62] Tanwar S, Rhodes F, Srivastava A, Trembling PM, Rosenberg WM. Inflammation and fibrosis in chronic liver diseases including non-alcoholic fatty liver disease and hepatitis C. World J Gastroenterol. 2020;26:109–33. 31969775 10.3748/wjg.v26.i2.109 PMC6962431

[63] Pei Q, Yi Q, Tang L. Liver fibrosis resolution: from molecular mechanisms to therapeutic opportunities. Int J Mol Sci. 2023;24:9671. 37298621 10.3390/ijms24119671 PMC10253436

[64] Altamirano-Barrera A, Barranco-Fragoso B, Méndez-Sánchez N. Management strategies for liver fibrosis. Ann Hepatol. 2017;16:48–56. 28051792 10.5604/16652681.1226814

[65] Lv K, Wang Y, Lou P, Liu S, Zhou P, Yang L, et al. Extracellular vesicles as advanced therapeutics for the resolution of organ fibrosis: current progress and future perspectives. Front Immunol. 2022;13:13. 10.3389/fimmu.2022.1042983 PMC9630482 36341339

[66] Tan Z, Sun H, Xue T, Gan C, Liu H, Xie Y, et al. Liver fibrosis: therapeutic targets and advances in Drug Therapy. Front Cell Dev Biol. 2021;9:2622. 10.3389/fcell.2021.730176 PMC8490799 34621747

[67] Liu P, Qian Y, Liu X, Zhu X, Zhang X, Lv Y, et al. Immunomodulatory role of mesenchymal stem cell therapy in liver fibrosis. Front Immunol. 2023;13: 1096402. 36685534 10.3389/fimmu.2022.1096402 PMC9848585

[68] Musiał-Wysocka A, Kot M, Majka M. The pros and cons of mesenchymal stem cell-based therapies. Cell Transpl. 2019;28:801. 10.1177/0963689719837897 PMC6719501 31018669

[69] Han C, Sun X, Liu L, Jiang H, Shen Y, Xu X et al. Exosomes and their therapeutic potentials of stem cells. Stem Cells Int. 2016;2016:7653489. 10.1155/2016/7653489 PMC4684885 26770213

[70] Rahimi B, Panahi M, Saraygord-Afshari N, Taheri N, Bilici M, Jafari D, et al. The secretome of mesenchymal stem cells and oxidative stress: challenges and opportunities in cell-free regenerative medicine. Mol Biol Rep. 2021;48:5607–19. 34191238 10.1007/s11033-021-06360-7

[71] Keshtkar S, Azarpira N, Ghahremani MH. Mesenchymal stem cell-derived extracellular vesicles: novel frontiers in regenerative medicine. Stem Cell Res Ther. 2018;9:1–9. 29523213 10.1186/s13287-018-0791-7 PMC5845209

[72] Ahmed L, Al-Massri K. New approaches for enhancement of the efficacy of mesenchymal stem cell-derived exosomes in cardiovascular diseases. Tissue Eng Regen Med. 2022;19(6):1129-1146. 10.1007/s13770-022-00469-x PMC9679101 35867309

[73] Tian S, Zhou X, Zhang M, Cui L, Li B, Liu Y, et al. Mesenchymal stem cell-derived exosomes protect against liver fibrosis via delivering miR-148a to target KLF6/STAT3 pathway in macrophages. Stem Cell Res Ther. 2022;13(1):330. 35858897 10.1186/s13287-022-03010-y PMC9297598

[74] Chen W, Lin F, Feng X, Yao Q, Yu Y, Gao F et al. MSC-derived exosomes attenuate hepatic fibrosis in primary sclerosing cholangitis through inhibition of Th17 differentiation. Asian J Pharm Sci. 2024;19(1):100889. 10.1016/j.ajps.2024.100889 PMC10900800 38419761

[75] Li T, Yan Y, Wang B, Qian H, Zhang X, Shen L, et al. Exosomes derived from human umbilical cord mesenchymal stem cells alleviate liver fibrosis. Stem Cells Dev. 2013;22:845–54. 23002959 10.1089/scd.2012.0395 PMC3585469

[76] Ohara M, Ohnishi S, Hosono H, Yamamoto K, Yuyama K, Nakamura H, et al. xtracellular vesicles from amnion‐derived mesenchymal stem cells ameliorate hepatic inflammation and fibrosis in rats. Stem Cells International. 2018;(1):3212643. 10.1155/2018/3212643 PMC6323530 30675167

[77] Kim J, Lee C, Shin Y, Wang S, Han J, Kim M, et al. sEVs from tonsil-derived mesenchymal stromal cells alleviate activation of hepatic stellate cells and liver fibrosis through miR-486-5p. Mol Ther. 2021;29:1471–86. 33348053 10.1016/j.ymthe.2020.12.025 PMC8058446

[78] Dong L, Pu Y, Chen X, Qi X, Zhang L, Xu L, et al. HUCMSC-extracellular vesicles downregulated hepatic stellate cell activation and reduced liver injury in S. japonicum-infected mice. Stem Cell Res Ther. 2020;11:1–11. 31918749 10.1186/s13287-019-1539-8 PMC6953150

[79] Tan Y, Huang Y, Mei R, Mao F, Yang D, Liu J, et al. HucMSC-derived exosomes delivered BECN1 induces ferroptosis of hepatic stellate cells via regulating the xCT/GPX4 axis. Cell Death Dis. 2022;13(4):1–11. 10.1038/s41419-022-04764-2 PMC8993870 35395830

[80] Wang S, Guo L, Ge J, Yu L, Cai T, Tian R, et al. Excess integrins cause lung entrapment of mesenchymal stem cells. Stem Cells. 2015;33:3315–26. 26148841 10.1002/stem.2087

[81] Jeong JO, Han JW, Kim JM, Cho HJ, Park C, Lee N, et al. Malignant tumor formation after transplantation of short-term cultured bone marrow mesenchymal stem cells in experimental myocardial infarction and Diabetic Neuropathy. Circ Res. 2011;108:1340–7. 21493893 10.1161/CIRCRESAHA.110.239848 PMC3109741

[82] Badillo AT, Beggs KJ, Javazon EH, Tebbets JC, Flake AW. Murine bone marrow stromal progenitor cells elicit an in vivo Cellular and Humoral Alloimmune Response. Biol Blood Marrow Transpl. 2007;13:412–22. 10.1016/j.bbmt.2006.12.447 PMC1892590 17382248

[83] Madrigal M, Rao KS, Riordan NH. A review of therapeutic effects of mesenchymal stem cell secretions and induction of secretory modification by different culture methods. J Transl Med. 2014;12:260. 10.1186/s12967-014-0260-8 PMC4197270 25304688

[84] Chen S, Sun F, Qian H, Xu W, Jiang J. Preconditioning and Engineering Strategies for Improving the Efficacy of Mesenchymal Stem Cell‐Derived Exosomes in Cell‐Free Therapy. Stem cells international. 2022;(1):1779346. 10.1155/2022/1779346 PMC9124131 35607400

[85] Zhang W, Liu R, Chen Y, Wang M, Du J. Crosstalk between Oxidative Stress and Exosomes. Oxid Med Cell Longev. 2022;3553617. 10.1155/2022/3553617 PMC9448575 36082080

[86] Wang J, Wu H, Peng Y, Zhao Y, Qin Y, Zhang Y, et al. Hypoxia adipose stem cell-derived exosomes promote high-quality healing of diabetic wound involves activation of PI3K/Akt pathways. J Nanobiotechnol. 2021;19:19. 10.1186/s12951-021-00942-0 PMC8261989 34233694

[87] Liu W, zhao, Ma Z, Li Jru, Kang X. Mesenchymal stem cell-derived exosomes: therapeutic opportunities and challenges for spinal cord injury. Stem Cell Res Ther. 2021;12(1):102. 33536064 10.1186/s13287-021-02153-8 PMC7860030

[88] Zhu L, Sun HT, Wang S, Huang SL, Zheng Y, Wang CQ, et al. Isolation and characterization of exosomes for cancer research. J Hematol Oncol. 2020;13:1-24. 10.1186/s13045-020-00987-y PMC7652679 33168028

[89] Cheng L, Zhang K, Wu S, Cui M, Xu T. Focus on mesenchymal stem cell‐derived exosomes: opportunities and challenges in cell‐free therapy. Stem cells international. 2017;(1):6305295. 10.1155/2017/6305295 PMC5749272 29410682

[90] Zhang Z, Dombroski JA, King MR. Engineering of exosomes to target cancer metastasis. Cell Mol Bioeng. 2020;13:1. 32030104 10.1007/s12195-019-00607-x PMC6981329

[91] Wang J, Bonacquisti EE, Brown AD, Nguyen J. Boosting the Biogenesis and Secretion of Mesenchymal Stem Cell-Derived exosomes. Cells. 2020;9. 10.3390/cells9030660 PMC7140620 32182815

[92] Antimisiaris SG, Mourtas S, Marazioti A. Exosomes and exosome-inspired vesicles for targeted drug delivery. Pharmaceutics. 2018;10. 30404188 10.3390/pharmaceutics10040218 PMC6321407

[93] Noronha Nc NDC, Mizukami A, Caliári-Oliveira C, Cominal JG, Rocha JLM, Covas DT et al. Priming approaches to improve the efficacy of mesenchymal stromal cell-based therapies. Stem Cell Res Ther. 2019;10(1):131. 10.1186/s13287-019-1224-y PMC6498654 31046833

[94] Lou G, Yang Y, Liu F, Ye B, Chen Z, Zheng M, et al. MiR-122 modification enhances the therapeutic efficacy of adipose tissue-derived mesenchymal stem cells against liver fibrosis. J Cell Mol Med. 2017;21:2963–73. 28544786 10.1111/jcmm.13208 PMC5661245

[95] Qu Y, Zhang Q, Cai X, Li F, Ma Z, Xu M, et al. Exosomes derived from mir-181-5p-modified adipose-derived mesenchymal stem cells prevent liver fibrosis via autophagy activation. J Cell Mol Med. 2017;21:2491–502. 28382720 10.1111/jcmm.13170 PMC5618698

[96] Wang J, Ye W, Jiang M, Zhou Y, Zheng J. Therapeutic potential of exosome derived from hepatocyte growth factor-overexpressing adipose mesenchymal stem cells in TGFβ1-stimulated hepatic stellate cells. Cytotechnology. 2024;76:217–29. 38495297 10.1007/s10616-023-00611-0 PMC10940570

[97] Xu AL, Han L, Yan J, Liu D, Wang W. Effects of mesenchymal stem cells-derived Extracellular vesicles on inhibition of hepatic fibrosis by delivering miR-200a. Tissue Eng Regen Med. 2024;21:609–24. 38568409 10.1007/s13770-024-00631-7 PMC11087440

[98] Cao Y, Yang H, Huang Y, Lu J, Du H, Wang B. Mesenchymal stem cell-derived exosomal miR-26a induces ferroptosis, suppresses hepatic stellate cell activation, and ameliorates liver fibrosis by modulating SLC7A11. Open Med (Warsaw, Poland). 2024;19:20240945. 10.1515/med-2024-0945 PMC11097046 38756248

[99] Fiore E, Domínguez LM, Bayo J, Malvicini M, Atorrasagasti C, Rodriguez M, et al. Human umbilical cord perivascular cells-derived extracellular vesicles mediate the transfer of IGF-I to the liver and ameliorate hepatic fibrogenesis in mice. Gene Ther. 2020;27:62–73. 31551525 10.1038/s41434-019-0102-7

[100] Zhu D, Sun Z, Wei J, Zhang Y, An W, Lin Y, et al. BMP7-Loaded human umbilical cord mesenchymal stem cell-derived small extracellular vesicles ameliorate liver fibrosis by targeting activated hepatic stellate cells. Int J Nanomed. 2024;19:3475–95. 10.2147/IJN.S450284 PMC11018131 38623080

[101] Ma L, Wei J, Zeng Y, Liu J, Xiao E, Kang Y, et al. Mesenchymal stem cell-originated exosomal circDIDO1 suppresses hepatic stellate cell activation by miR-141-3p/PTEN/AKT pathway in human liver fibrosis. Drug Deliv. 2022;29:440. 10.1080/10717544.2022.2030428 PMC8812765 35099348

[102] Haney MJ, Klyachko NL, Zhao Y, Gupta R, Plotnikova EG, He Z, et al. Exosomes as drug delivery vehicles for Parkinson’s disease therapy. J Control Release. 2015;207:18–30. 25836593 10.1016/j.jconrel.2015.03.033 PMC4430381

[103] Walker S, Busatto S, Pham A, Tian M, Suh A, Carson K, et al. Extracellular vesicle-based drug delivery systems for cancer treatment. Theranostics. 2019;9:8001–17. 31754377 10.7150/thno.37097 PMC6857056

[104] Woo CH, Kim HK, Jung GY, Jung YJ, Lee KS, Yun YE, et al. Small extracellular vesicles from human adipose-derived stem cells attenuate cartilage degeneration. J Extracell Vesicles. 2020;9(1):1735249. 10.1080/20013078.2020.1735249 PMC7144299 32284824

[105] Liang L, Zhao L, Wang Y, Wang Y. Treatment for hepatocellular carcinoma is enhanced when Norcantharidin is encapsulated in Exosomes derived from bone marrow mesenchymal stem cells. Mol Pharm. 2021;18:1003–13. 33527831 10.1021/acs.molpharmaceut.0c00976

[106] Xing H, Liang C, Xu X, Sun H, Ma X, Jiang Z. Mesenchymal stroma/stem-like cells of GARP knockdown inhibits cell proliferation and invasion of mouse colon cancer cells (MC38) through exosomes. J Cell Mol Med. 2020;24:13984–90. 33155413 10.1111/jcmm.16008 PMC7753840

[107] Gomari H, Moghadam MF, Soleimani M, Ghavami M, Khodashenas S. Targeted delivery of doxorubicin to HER2 positive tumor models. Int J Nanomed. 2019;14:5679–90. 10.2147/IJN.S210731 PMC6662522 31413568

[108] Wu P, Zhang B, Ocansey DKW, Xu W, Qian H. Extracellular vesicles: a bright star of nanomedicine. Biomaterials. 2021;269:120467. 10.1016/j.biomaterials.2020.120467 33189359

[109] Guo S, Perets N, Betzer O, Ben-Shaul S, Sheinin A, Michaelevski I, et al. Intranasal delivery of mesenchymal stem cell derived exosomes loaded with phosphatase and tensin homolog siRNA repairs complete spinal cord Injury. ACS Nano. 2019;13:10015–28. 31454225 10.1021/acsnano.9b01892

[110] Tang M, Chen Y, Li B, Sugimoto H, Yang S, Yang C et al. Therapeutic targeting of STAT3 with small interference RNAs and antisense oligonucleotides embedded exosomes in liver fibrosis. FASEB J. 2021;35(5):e21557. 10.1096/fj.202002777RR PMC10851328 33855751

[111] Tang M, Guo C, Sun M, Zhou H, Peng X, Dai J, et al. Effective delivery of osteopontin small interference RNA using exosomes suppresses liver fibrosis via TGF-β1 signaling. Front Pharmacol. 2022;13:13. 10.3389/fphar.2022.882243 PMC9478741 36120332

[112] Zhou W, Zhou Y, Chen X, Ning T, Chen H, Guo Q et al. Pancreatic cancer-targeting exosomes for enhancing immunotherapy and reprogramming tumor microenvironment. Biomaterials. 2021;268:120546. 10.1016/j.biomaterials.2020.120546 33253966

[113] Greco KA, Franzen CA, Foreman KE, Flanigan RC, Kuo PC, Gupta GN. PLK-1 Silencing in Bladder Cancer by siRNA delivered with exosomes. Urology. 2016;91:241.e1-241.e7. 26876462 10.1016/j.urology.2016.01.028

[114] Shojaei S, Hashemi SM, Ghanbarian H, Salehi M, Mohammadi-Yeganeh S. Effect of mesenchymal stem cells-derived exosomes on tumor microenvironment: tumor progression versus tumor suppression. J Cell Physiol. 2019;234:3394–409. 30362503 10.1002/jcp.27326

[115] Azizsoltani A, Hatami B, Zali MR, Mahdavi V, Baghaei K, Alizadeh E. Obeticholic acid-loaded exosomes attenuate liver fibrosis through dual targeting of the FXR signaling pathway and ECM remodeling. Biomed Pharmacother. 2023;168: 115777. 37913732 10.1016/j.biopha.2023.115777

[116] Fang J, Liang W. ASCs -derived exosomes loaded with vitamin A and quercetin inhibit rapid senescence-like response after acute liver injury. Biochem Biophys Res Commun. 2021;572:125–30. 34364291 10.1016/j.bbrc.2021.07.059

[117] Ashour AA, El-Kamel AH, Mehanna RA, Mourad G, Heikal LA. Luteolin-loaded exosomes derived from bone marrow mesenchymal stem cells: a promising therapy for liver fibrosis. Drug delivery. 2022;29(1):3270–80. 36330597 10.1080/10717544.2022.2142700 PMC9639476

[118] Zhai Z, Cui T, Chen J, Mao X, Zhang T. Advancements in engineered mesenchymal stem cell exosomes for chronic lung disease treatment. J Transl Med. 2023;21(1):895. 10.1186/s12967-023-04729-9 PMC10709966 38071321

[119] Xu M, Feng T, Liu B, Qiu F, Xu Y, Zhao Y, et al. Engineered exosomes: desirable target-tracking characteristics for cerebrovascular and neurodegenerative disease therapies. Theranostics. 2021;11:8926–44. 34522219 10.7150/thno.62330 PMC8419041

[120] Mentkowski KI, Snitzer JD, Rusnak S, Lang JK. Therapeutic potential of engineered extracellular vesicles. AAPS J. 2018;20:50. 29546642 10.1208/s12248-018-0211-z PMC8299397

[121] Villata S, Canta M, Cauda V. EVs and bioengineering: from cellular products to Engineered Nanomachines. Int J Mol Sci. 2020;21:1–32. 10.3390/ijms21176048 PMC7504061 32842627

[122] Salunkhe S, Dheeraj, Basak M, Chitkara D, Mittal A. Surface functionalization of exosomes for target-specific delivery and in vivo imaging & tracking: strategies and significance. J Control Release. 2020;326:599–614. 10.1016/j.jconrel.2020.07.042 32730952

[123] Lin Y, Yan M, Bai Z, Xie Y, Ren L, Wei J, et al. Huc-MSC-derived exosomes modified with the targeting peptide of aHSCs for liver fibrosis therapy. J Nanobiotechnol. 2022;20:20. 10.1186/s12951-022-01636-x PMC9526331 36183106

[124] Hung ME, Leonard JN. Stabilization of exosome-targeting peptides via engineered glycosylation. J Biol Chem. 2015;290:8166–72. 25657008 10.1074/jbc.M114.621383 PMC4375473

[125] Tamura R, Uemoto S, Tabata Y. Augmented liver targeting of exosomes by surface modification with cationized pullulan. Acta Biomater. 2017;57:274–84. 28483695 10.1016/j.actbio.2017.05.013

[126] Kooijmans SAA, Fliervoet LAL, Van Der Meel R, Fens MHAM, Heijnen HFG, Van Bergen En Henegouwen PMP, et al. PEGylated and targeted extracellular vesicles display enhanced cell specificity and circulation time. J Control Release. 2016;224:77–85. 26773767 10.1016/j.jconrel.2016.01.009

[127] Hwang DW, Jo MJ, Lee JH, Kang H, Bao K, Hu S et al. Chemical modulation of bioengineered exosomes for tissue-specific Biodistribution. Adv Ther. 2019;2(11):1900111. 10.1002/adtp.201900111 PMC7172497 32318623

[128] Nakase I, Noguchi K, Aoki A, Takatani-Nakase T, Fujii I, Futaki S. Arginine-rich cell-penetrating peptide-modified extracellular vesicles for active macropinocytosis induction and efficient intracellular delivery. Sci Rep. 2017;7:7. 28512335 10.1038/s41598-017-02014-6 PMC5434003

[129] Zheng L, Gong H, Zhang J, Guo L, Zhai Z, Xia S et al. Strategies to improve the therapeutic efficacy of mesenchymal stem cell-derived extracellular vesicle (MSC-EV): a promising cell-free therapy for liver disease. Front Bioeng Biotechnol. 2023;11:1322514. 10.3389/fbioe.2023.1322514 PMC10753838 38155924

[130] Qu Q, Fu B, Long Y, Liu Z-Y, Tian X-H. Current strategies for promoting the large-scale production of Exosomes. Curr Neuropharmacol. 2023;21:1964–79. 36797614 10.2174/1570159X21666230216095938 PMC10514529

[131] Gómez-Ferrer M, Villanueva-Badenas E, Sánchez-Sánchez R, Sánchez-López CM, Baquero MC, Sepúlveda P et al. HIF-1α and pro-inflammatory signaling improves the Immunomodulatory activity of MSC-Derived Extracellular vesicles. Int J Mol Sci. 2021;22(7):3416. 10.3390/ijms22073416 PMC8036951 33810359

[132] Hassan MNF, Bin, Yazid MD, Yunus MHM, Chowdhury SR, Lokanathan Y, Idrus RBH, et al. Large‐scale expansion of human mesenchymal stem cells. Stem Cells International. 2020;(1):9529465. 10.1155/2020/9529465 PMC7378617 32733574

[133] Yan L, Wu X. Exosomes produced from 3D cultures of umbilical cord mesenchymal stem cells in a hollow-fiber bioreactor show improved osteochondral regeneration activity. Cell Biol Toxicol. 2020;36:165–78. 31820164 10.1007/s10565-019-09504-5 PMC7196084

[134] Wang N, Li X, Zhong Z, Qiu Y, Liu S, Wu H, et al. 3D hESC exosomes enriched with mir-6766-3p ameliorates liver fibrosis by attenuating activated stellate cells through targeting the TGFβRII-SMADS pathway. J Nanobiotechnol. 2021;19:19. 10.1186/s12951-021-01138-2 PMC8686281 34930304

[135] Xiong Y, Tang R, Xu J, Jiang W, Gong Z, Zhang L et al. Sequential transplantation of exosomes and mesenchymal stem cells pretreated with a combination of hypoxia and Tongxinluo efficiently facilitates cardiac repair. Stem Cell Res Ther. 2022;13(1):63. 10.1186/s13287-022-02736-z PMC8822662 35130979

[136] Wei X, Zheng W, Tian P, Liu H, He Y, Peng M, et al. Administration of glycyrrhetinic acid reinforces therapeutic effects of mesenchymal stem cell-derived exosome against acute liver ischemia-reperfusion injury. J Cell Mol Med. 2020;24:11211–20. 32902129 10.1111/jcmm.15675 PMC7576231

[137] Kharaziha P, Hellström PM, Noorinayer B, Farzaneh F, Aghajani K, Jafari F, et al. Improvement of liver function in liver cirrhosis patients after autologous mesenchymal stem cell injection: a phase I-II clinical trial. Eur J Gastroenterol Hepatol. 2009;21:1199–205. 19455046 10.1097/MEG.0b013e32832a1f6c

[138] Liang J, Zhang H, Zhao C, Wang D, Ma X, Zhao S, et al. Effects of allogeneic mesenchymal stem cell transplantation in the treatment of liver cirrhosis caused by autoimmune diseases. Int J Rheum Dis. 2017;20:1219–26. 28217916 10.1111/1756-185X.13015

[139] Vosough M, Moossavi S, Mardpour S, Akhlaghpoor S, Azimian V, Jarughi N, et al. Repeated intraportal Injection of mesenchymal stem cells in combination with pioglitazone in patients with compensated cirrhosis: a clinical report of two cases. Arch Iran Med. 2016;19:131–6. 26838084

[140] Lotfy A, AboQuella NM, Wang H. Mesenchymal stromal/stem cell (MSC)-derived exosomes in clinical trials. Stem Cell Res Ther. 2023;14:1–18. 37024925 10.1186/s13287-023-03287-7 PMC10079493

[141] Kordelas L, Rebmann V, Ludwig AK, Radtke S, Ruesing J, Doeppner TR, et al. MSC-derived exosomes: a novel tool to treat therapy-refractory graft-versus-host disease. Leukemia. 2014;28:970–3. 24445866 10.1038/leu.2014.41

[142] Nassar W, El-Ansary M, Sabry D, Mostafa MA, Fayad T, Kotb E, et al. Umbilical cord mesenchymal stem cells derived extracellular vesicles can safely ameliorate the progression of chronic kidney diseases. Biomater Res. 2016;20:20. 27499886 10.1186/s40824-016-0068-0 PMC4974791

[143] Kwon HH, Yang SH, Lee J, Park BC, Park KY, Jung JY, et al. Combination treatment with human adipose tissue stem cell-derived exosomes and fractional CO2 laser for Acne scars: a 12-week prospective, Double-blind, Randomized, Split-face study. Acta Derm Venereol. 2020;100:1–8. 10.2340/00015555-3666 PMC9309822 33073298

[144] Sengupta V, Sengupta S, Lazo A, Woods P, Nolan A, Bremer N. Exosomes derived from bone marrow mesenchymal stem cells as treatment for severe COVID-19. Stem Cells Dev. 2020;29:747–54. 32380908 10.1089/scd.2020.0080 PMC7310206

[145] Xu Y, Zhou X, Wang X, Jin Y, Zhou L, Ye J. Progress of mesenchymal stem cells (MSCs) & MSC-Exosomes combined with drugs intervention in liver fibrosis. Biomed Pharmacother. 2024;176:116848. 10.1016/j.biopha.2024.116848 38834005

[146] Sun Y, Liu G, Zhang K, Cao Q, Liu T, Li J. Mesenchymal stem cells-derived exosomes for drug delivery. Stem Cell Res Ther. 2021;12:12. 34717769 10.1186/s13287-021-02629-7 PMC8557580

[147] Liu A, Lin D, Zhao H, Chen L, Cai B, Lin K, et al. Optimized BMSC-derived osteoinductive exosomes immobilized in hierarchical scaffold via lyophilization for bone repair through Bmpr2/Acvr2b competitive receptor-activated smad pathway. Biomaterials. 2021;272:120467. 10.1016/j.biomaterials.2021.120718 33838528

[148] Driscoll J, Yan IK, Patel T. Development of a Lyophilized off-the-shelf mesenchymal stem cell-derived Acellular therapeutic. Pharmaceutics. 2022;14(4):849. 10.3390/pharmaceutics14040849 PMC9030800 35456683

[149] Guo Y, Zhai Y, Wu L, Wang Y, Wu P, Xiong L. Mesenchymal stem cell-derived extracellular vesicles: pleiotropic impacts on breast Cancer occurrence, Development, and Therapy. Int J Mol Sci. 2022;23(6):2927. 10.3390/ijms23062927 PMC8954385 35328347

[150] Harrell CR, Jankovic MG, Fellabaum C, Volarevic A, Djonov V, Arsenijevic A, et al. Molecular mechanisms responsible for anti-inflammatory and immunosuppressive effects of mesenchymal stem cell-derived factors. Adv Exp Med Biol. 2019;1084:187–206. 31175638 10.1007/5584_2018_306

[151] Adem B, Melo SA. Animal models in exosomes research: what the future holds. Nov implic exosomes Diagn Treat Cancer Infect Dis. 2017;53.

[152] Benten D, Kluwe J, Wirth JW, Thiele ND, Follenzi A, Bhargava KK, et al. A humanized mouse model of liver fibrosis following expansion of transplanted hepatic stellate cells. Lab Invest. 2018;98:525–36. 29352225 10.1038/s41374-017-0010-7 PMC6526950

